# Environmental transcriptomes of invasive dreissena, a model species in ecotoxicology and invasion biology

**DOI:** 10.1038/s41597-019-0252-x

**Published:** 2019-10-25

**Authors:** Romain Péden, Pascal Poupin, Bénédicte Sohm, Justine Flayac, Laure Giambérini, Christophe Klopp, Fanny Louis, Sandrine Pain-Devin, Marine Potet, Rémy-Félix Serre, Simon Devin

**Affiliations:** 1 Université de Lorraine, CNRS, LIEC, F-57000 Metz, France; 2 Université Reims Champagne Ardenne, UMR-I 02 SEBIO, 51097 Reims, France; 3 Plate-forme bio-informatique Genotoul, Mathématiques et Informatique Appliquées de Toulouse, INRA, 31326 Castanet-Tolosan, France; 4 INRA, US 1426, GeT-PlaGe, Genotoul, INRA Auzeville, Castanet Tolosan, Cedex France

**Keywords:** Transcriptomics, Molecular ecology

## Abstract

Dreissenids are established model species for ecological and ecotoxicological studies, since they are sessile and filter feeder organisms and reflect *in situ* freshwater quality. Despite this strong interest for hydrosystem biomonitoring, omics data are still scarce. In the present study, we achieved full *de novo* assembly transcriptomes of digestive glands to gain insight into *Dreissena polymorpha* and *D*. *rostriformis bugensis* molecular knowledge. Transcriptomes were obtained by Illumina RNA sequencing of seventy-nine organisms issued from fifteen populations inhabiting sites that exhibits multiple freshwater contamination levels and different hydrosystem topographies (open or closed systems). Based on a recent *de novo* assembly algorithm, we carried out a complete, quality-checked and annotated transcriptomes. The power of the present study lies in the completeness of transcriptomes gathering multipopulational organisms sequencing and its full availability through an open access interface that gives a friendly and ready-to-use access to data. The use of such data for proteogenomic and targeted biological pathway investigations purpose is promising as they are first full transcriptomes for this two *Dreissena* species.

## Abstract (summary)

**Table:** 

Measurement(s)	transcriptome
Technology Type(s)	RNA sequencing
Factor Type(s)	sampling location
Sample Characteristic - Organism	Dreissena polymorpha • Dreissena rostriformis bugensis
Sample Characteristic - Environment	freshwater biome
Sample Characteristic - Location	Metropolitan France

Machine-accessible metadata file describing the reported data: 10.6084/m9.figshare.9934226

## Background & Summary

*Dreissena polymorpha* and *D*. *rostriformis bugensis*, also known as the zebra and the quagga mussels, are model species in ecology and ecotoxicology since the early eighties. These invasive species are now commonly found in freshwaters of the northern hemisphere. *D*. *polymorpha* belongs to the 100 of the World’s Worst Invasive Alien Species identified by the IUCN (http://www.iucngisd.org/gisd/100_worst.php). It spread from Ponto-Caspian basin to northern and western Europe in the 19th century, and to North America and Middle East (Turkey) in the late 20th century, while *D*. *r*. *bugensis* colonized both North America and Western Europe between the late 20th and at the beginning of the 21th century^1,2^. They can reach high densities in invaded ecosystems^3^, inducing important ecological and economic damages^4,5^. Their distribution and invasion dynamic is well documented, as well as ecological features of colonized ecosystems. Dreissenids thus become model species in ecology of biological invasion to identify pathway of invasion, and genetic mechanisms associated to colonization at various spatial and temporal scales.

Their ability to tolerate a wide range of environmental contaminants and their presence in almost every major hydrosystem in Europe and North America also lead dreissenids to become a model species for ecotoxicologists, who identified them as the counterpart of *Mytilus* for freshwaters^6^. Indeed, being abundant, sessile, filter feeder, mussels bioaccumulate contaminants present in the water column, and are good candidates to be included in biomonitoring programs. It allows to evaluate both the presence of contaminants through bioaccumulation measurements^7,8^ and their effects through the use of biomarkers, either in the field or in laboratory^9–11^.

**Fig. 1:**
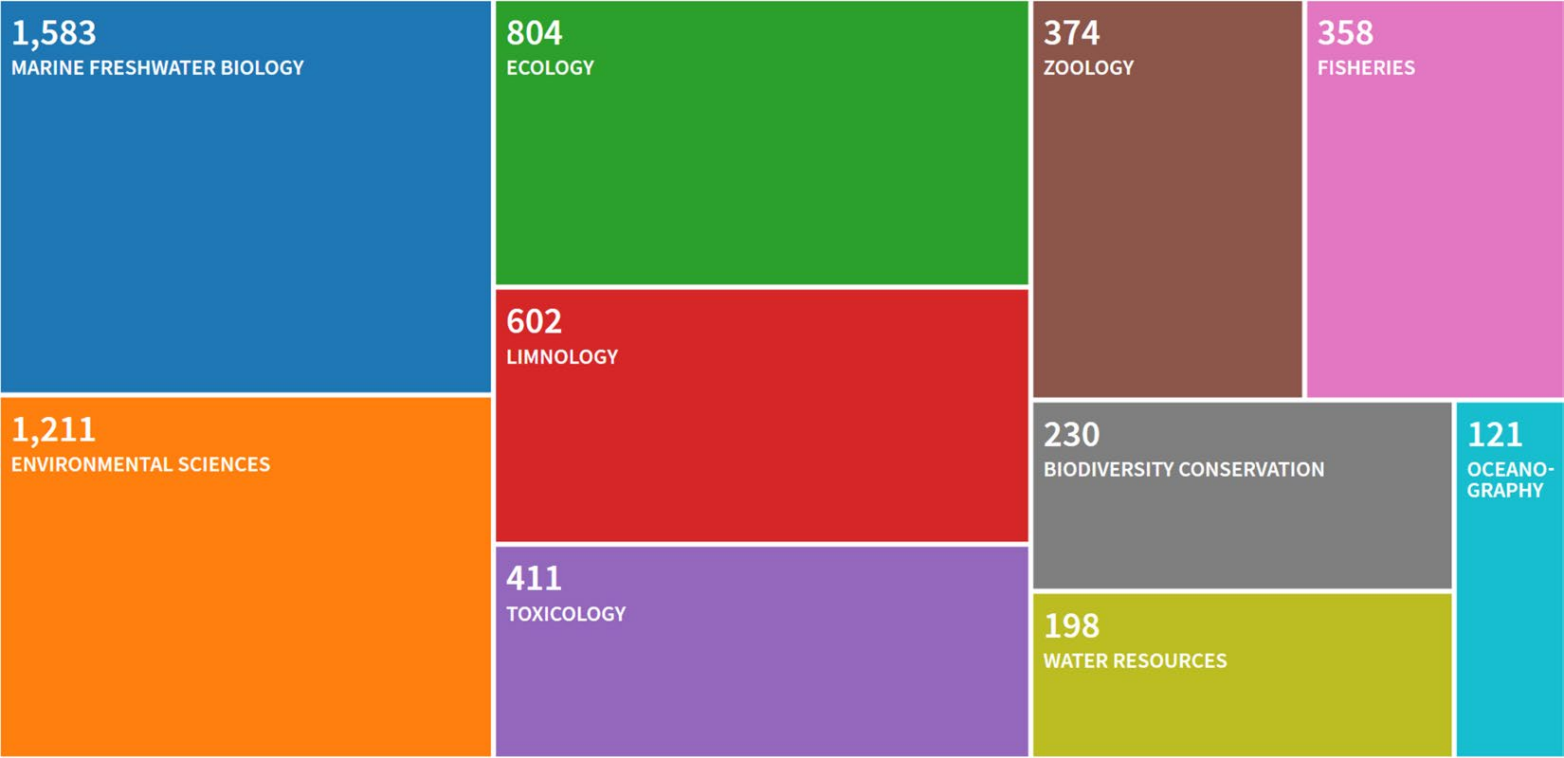
Top 10 fields treemap for *Dreissena* publications.

A bibliographic research with the keyword “*dreissena*” performed on the Web of Science the 22th of january 2019 reveals that 3,667 articles were published since 1990, with *ca*. 180 article each year since 2010. In the meantime, these articles were cited 97,000 times, with a h-index of 119. Their distribution among scientific topics (Fig. 1) reveals this dual interest in ecology and ecotoxicology. However, over the eleven Bioprojects present in NCBI for *Dreissena*, most focused on foot, gill or mantle tissues where few concern digestive glands. Moreover, the majority of their studies are not *de novo* RNA-seq but micro-array studies and almost none of them made sequences available in accessible repositories. In the present study, digestive gland was chosen for its importance in ecotoxicology due to its central roles in detoxication functions and energy metabolism^12^.

Indeed, genetic studies on zebra and quagga mussel mainly concerns their spread dynamic through a population genetic perspective^13–15^, the identification of genetic markers for their identification, and bivalve phylogeny and phylogeography^13,16–19^. When the focus of the study was to elucidate toxic effect pathways, only a very limited set of genes were targeted, corresponding to the main processes implied in organism’s response to contaminant^20,21^.

Studies from several populations of the two species can pave the way for several new research possibilities. The most promising and useful ones are to make available a reference transcriptome for proteogenomic studies, to identify sequences to develop new sets of biomarkers, and to better understand acclimation mechanisms occuring during the invasion process and implied in tolerance to contamination.

## Methods

### Sampling and RNA extraction

Samples were performed on 12 sites representative of french dreissenid populations diversity. Sites were chosen according to their variable contamination levels, chemicophysical quality and topography characteristics (see Supplementary Table 1). Five bivalves were sampled by populations and digestive glands were *in situ* dissected and stored in RNAlater (Qiagen) at 4 °C. Total RNA extraction was done using RNeasy MinElute kit (Qiagen) according to the manufacturer’s protocol with slight modifications. Briefly, tissus disruption was done first with a pestle and then with a beads beater with glass bead (200 µm) for 1 min. at max speed in 1 mL of RLT buffer supplemented with 40 mM DTT. Samples were centrifuged 3 min at 20,000 × g. Qiagen protocol was used for the next steps. Genomic DNA was removed by DNase digestion on column and total RNA was eluted in ultra pure water. RNA purity and quantity were assessed by OD measurements (OD 260 nm and OD ratio 260/280 and 260/230) and RNA integrity was checked using Bioanalyseur 2100 (Agilent, CA, USA). Total RNA samples were stored at −80. Samples were send to GeT-PlaGe core facility in dried-ice.

### RNA sequencing

RNAseq was performed at the GeT-PlaGe core facility, INRA Toulouse. RNA-seq libraries have been prepared according to Illumina’s protocols using the Illumina TruSeq Stranded mRNA sample prep kit to analyze mRNA. The 79 individuals were sequenced separately on 8 different lanes of one flow cell. Briefly, mRNA were selected using poly-T beads. Then, RNA were fragmented to generate double stranded cDNA and adaptators were ligated to be sequenced. Eleven cycles of PCR were applied to amplify libraries. Library quality was assessed using a Fragment Analyser and libraries were quantified by QPCR using the Kapa Library Quantification Kit. RNA-seq experiments have been performed on an Illumina HiSeq3000 using a paired-end read length of 2 × 150 pb with the Illumina HiSeq3000 sequencing kits.

### *De novo* assembly and annotation

The RNA-seq libraries read quality was evaluated using FastQC (http://www.bioinformatics.babraham.ac.uk/projects/fastqc). Reads from 45 *D*. *polymorpha* and 34 *D*. *rostriformis bugensis* individuals were cleaned, filtered and *de novo* assembled for each species with DRAP (*De novo* RNA-seq Assembly Pipeline, version 1.7)^22^ using the Oases assembler^23^. Contigs were kept if they had at least one FPKM. Contigs were then aligned with NCBI BLAST (version 2.2.26, e-value under 1e-5 parameter) on Refseq, Swissprot and three databases issued from Ensembl (*Crassostrea gigas*, *Lottia gigantea* and *Lingula anatina* sequences) to retrieve corresponding annotations. Contigs were also processed with RNAmmer (version 1.2, standard parameters)^24^ to find ribosomal genes, with RepeatMasker (version open-4-0-3, -engine crossmatch -gccalc -species *Crassostrea gigas* parameters, http://www.repeatmasker.org) to list contained repeats and with InterProScan (version 4.8, -goterms -pathways parameters)^25^ for gene ontology and structural annotation. Reads were realigned back to contigs with BWA (version 0.7.12, standard parameters, mem algorithm)^26^. The resulting sam files were compressed, sorted and indexed with SAMtools (view, sort and index programs, version 1.1, standard parameters)^27^. Contig expression counts were generated from the bam files with SAMtools (IdxStats program, version 1.1, standard parameters) and merged with UNIX commands (cut, paste). Alignment files were then filtered for duplicates with SAMtools (rmdup program, version 1.1, standard parameters) before variant calling (SNPs and INDELs). Resulting bam files were processed with GATK (version v3.0–0-g6bad1c6, -glm BOTH parameter) following the best practices found on the GATK website^28^. All the results were uploaded in a RNAbrowse instance^29^ and can be accessed from the web at http://ngspipelines.toulouse.inra.fr:9014/. Further KEGG annotation were done with KAAS interface (KEGG Automatic Annotation Server) using contig sequences in fasta format.

Biomarker explorations were done using the implemented BLAST in NGSpipeline interface. Biomarker sequences come from close related species (*i*.*e*. molluscs). Candidates were selected by the user on score, e-values, identity and length. If several candidates exist, the sequence with highest score was selected.

## Data Records

Raw reads and assemblies were gathered in the same NCBI BioProject (PRJNA507340) which includes all BioSamples used for transcriptome assembly (Table 1)^30–32^. All datasets were also available online on sequencing platform web interface (Table 2)^33^. Datasets annotations (contigs, KEGG, GO) are available on Figshare (Table 3)^34^.

**Table 1:** BioProject deposit. The BioProject gathered all BioSamples, SRA^30^ and TSA^31,32^ related to this Data Descriptor.

BioProject	Datasets (nb)	Accessions
PRJNA507340	BioSamples (79)	SAMN10537936 to SAMN10538014
	SRA (79)	SRR8354718 to SRR8354796
	TSA (2)	GHIW00000000 and GHIX00000000

**Table 2:** NGSPipeline deposit.

Data type	URL	Implemented softwares
Web interface	http://ngspipelines.toulouse.inra.fr:9014	BioMart and BLAST

Datasets are fully accessible in an user friendly web interface provided by Genotoul sequencing and bioinformatic platform^29,33^. Biomart and BLAST are implemented for quick database interrogation.

**Table 3:** Figshare deposit.

Descriptive filename	Data format
*Dreissena_polymorpha*_contig_sequences	fasta
*Dreissena_rostriformis_bugensis*_contig_sequences	fasta
BLAST_annotations_for_*Dreissena_polymorpha*_contigs	csv
BLAST_annotations_for_*Dreissena_rostriformis_bugensis*_contigs	csv
GOterms_annotations_for_*Dreissena_polymorpha*_contigs	csv
GOterms_annotations_for_*Dreissena_rostriformis_bugensis*_contigs	csv
KEGG_annotations_for_*Dreissena_polymorpha*_contigs	csv
KEGG_annotations_for_*Dreissena_rostriformis_bugensis*_contigs	csv

Supplementary files are available on figshare including annotations (GO, KEGG) and ready to use transcriptomes in fasta format^34^.

## Technical Validation

### Extraction and RNA integrity

Total RNA purity was assessed with a Nanodrop ND-100 Spectrophotometer (Nanodrop Technologies, Wilmington, USA) and RNA with a 260/280 and 260/230 ratio superior to 1.8 were kept. RNA integrity was evaluated with a Bioanalyzer (Agilent RNA 6000 Nano kit). Due to a non conventional 18S/28S ribosomal ratio in bivalve, sample quality was evaluated with the 18S/28S ratio and on the electropherogram (Supplementary Fig. S1). The absence of degradation fragment in the 5S, Fast and Inter regions was a criteria of selection for sequencing.

### *De novo* transcriptome assembly validation

A total of 94,217 contigs were assembled spanning from 200 to 40,000 bp and with an average length of 2,314 bp for *D*. *polymorpha* and 1,972 for *D*. *r*. *bugensis* (Table 4). Assembly validation aims at verifying the correspondence between contigs and assembled reads, between contigs and the proteome of a phylogenetically related species and to check if the contigs host single copy awaited proteins. First, reads were mapped back to contigs for each sample in order to monitor the realignment rates revealing a realignment rate above 95% for both species (Table 5). Second, *Crassostrea gigas* proteins were aligned to contigs using BLAT (v. 35 × 1)^35^. Proteins mapping at 50% identity and over 50% of the protein length were counted to measure the share of proteins correctly reconstructed by the assembly (Table 6). Last, contigs were processed with BUSCO version 3.0.2^36^ using the metazoa OrthoDB (v. 9) database to check for awaited proteins (Table 7) which shows that almost 95% of expected metazoa BUSCO sequences were found as complete in our *de novo* transcriptome.

**Table 4:** Assembly metrics.

Type	*D*. *polymorpha*	*D*. *r*. *bugensis*
Number of contigs	44,538	49,679
Total size of contigs (bp)	103,039,811	97,982,186
N50 (bp)	3,094	2,674
Average length (bp)	2,314	1,972
Longest contig (bp)	39,481	39,593
Shortest contig (bp)	210	207

**Table 5:** Individual realignment statistics.

Type	*D*. *polymorpha*		*D*. *r*. *bugensis*	
Total read count average	69,815,940	±9,645,652	73,730,343	±9,641,714
Alignment rate	97.56%	±0.7%	98.15%	±0.05%
Properly paired rate	88.84%	±1.15%	86.58%	±1.60%

**Table 6:** *Crassostrea gigas* proteins.

Type	*D*. *polymorpha*	*D*. *r*. *bugensis*
Number of *C*. *gigas* proteins matching	4,352	4,281
Number of unique Dreissenid contigs mapped to	3,594	3,558

**Table 7:** BUSCO analysis.

Type	*D*. *polymorpha*		*D*. *r*. *bugensis*	
Complete BUSCOs	921	94.2%	923	94.4%
Complete and single-copy BUSCOs	494	50.5%	450	46.0%
Complete and duplicated BUSCOs	427	43.7%	473	48.4%
Fragmented BUSCOs	5	0.5%	7	0.7%
Missing BUSCOs	52	5.3%	48	4.9%
Total BUSCO groups searched	978	100%	978	100%

### Annotation quality

**Fig. 2:**
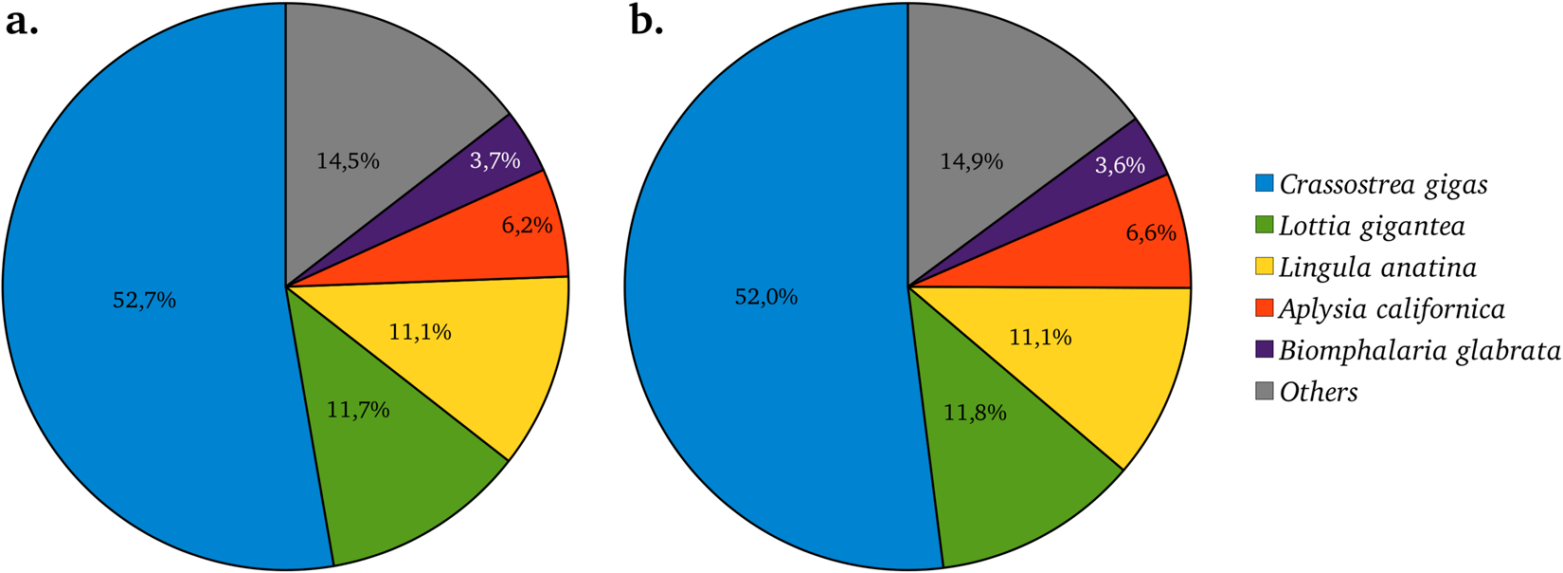
Most representated species hits. Top 5 best species hits for (**A**) *Dreissena polymorpha* and (**B**). *Dreissena rostriformis bugensis*.

More than the half of assembled contigs found annotation with an e-value under 1e-5 (59% of 44,538 *D*. *polymorpha* contigs and 57% of 49,679 *D*. *rostriformis bugensis* contigs). Among top 5 species found as best annotation, *Crassostrea gigas* represent the best hit species matching with approximately 53% of *D*. *polymorpha* and *D*. *r*. *bugensis* sequences (Fig. 2). Among the “others” category, some non bivalves sequences can be present thanks to the analyzed tissue (as digestive gland may contain processing foods). Sequence functional annotations successfully attribute GOterms to 35,4% and KEGG to 21.6% of *D*. *polymorpha* contigs and attribute GOterms to 34,2% and KEGG to 20.6% of *D*. *r*. *bugensis* contigs. Full contigs annotation as well as functional annotations are available in the figshare deposit in csv format for quick and easy reuse (Table 3).

## Usage Notes

We present here the first whole *de novo* transcriptomes of the two invasive *Dreissena polymorpha* and *D*. *rostriformis bugensis* species who will be fully available in TSA repository. One of the strength of proposed transcriptome is the conditions in which it was obtained: by investigating individuals coming from several populations, inhabiting contrasted hydrosystems, we encompassed as much as possible the variability of the transcriptome induced by environmental conditions (pollution, biotic interactions, physico-chemistry, climate…), with a mRNA set that is representative of this ecological heterogenity. Our ambitious approach also lead to identification of numerous sequence variants (insertion, deletion and single-nucleotide polymorphism), since studied populations are established for a long time and may have been prone to mutation.

In ecotoxicology, biomarkers implied in responses to oxidative stress, heat shock or xenobiotic exposure are used as indicators of stress. Nowadays, multibiomarker approaches have become a standard, considering that the diversity of contaminants that organisms are exposed to can generate a wide diversity of biological responses. Here, we first wanted to focus on the most frequently used biomarkers in ecotoxicology and which are listed in Table 8. Biomarkers were screened, manually checked and all were found expressed in our both species digestive glands. This table also shows the closest species hits (*blastn*) and biomarkers selected here exhibit relatively high value of homology with the corresponding sequence in other mollusk, supporting our contig assignations. The high sequence conservation levels observed here may facilitate the development and adaptation of further measurements assays from existing assays in close related species. Interspecies sequence alignments were also investigated indicating a high levels of homologies between the two species (Table 9). In the same way, this may lead to the development of biomarker based assays that, because of sequence similarity, could be cross compatible for *D*. *polymorpha* and *D*. *r*. *bugensis*.

**Table 8:** Biomarker best contig hits and closest species hits.

Biomarkers	Contig database name			Closest blastn hit			
				E-value		% Identity	
	*D*. *polymorpha*	*D*. *r*. *bugensis*	Species	*D*. *p*.	*D*. *r*. *b*.	*D*. *p*.	*D*. *r*. *b*.
Acetylcholinesterase	Dp_LOC105324424.2.2	Db_LOC105324424.3.3	*Crassostrea virginica*	2e-8	4e-12	68%	67%
Beta Actine	Dp_ACT.1.3	Db_ACT.2.4	*Crassostrea virginica*	0.0	0.0	90%	90%
Catalase	Dp_LOC105339902	Db_LOC105339902.2.4	*Corbicula fluminae*	0.0	0.0	77%	76%
Superoxide dismutase (Cu-Zn)	Dp_SODC	Db_SODC	*Ostrea edulis*	4e-55	1e-48	73%	72%
Superoxide dismutase (Mn)	Dp_LOC101852344	Db_LOC101852344	*Haliotis fulgens*	3e-78	3e-77	72%	72%
Estrogen receptor	Dp_LOC105318922.2.2	Db_LOC105318922.1.2	*Ruditapes philippinarum*	0.0	0.0	75%	74%
GABARAP	Dp_LOC105335545.1.2	Db_LOC105335545.1.6	*Meretrix meretrix*	2e-103	1e-98	84%	83%
Glutathione peroxidase 1	Dp_GPX1.1.2	Db_GPX1.1.2	*Ruditapes philippinarum*	1e-54	6e-59	72%	73%
Glutathione peroxidase (Se)	Dp_LOC106070504.1.5	Db_LOC106070504.6.8	*Meretrix meretrix*	3e-79	6e-75	73%	73%
Heat Shock Protein70	Dp_HSP7D.3.8	Db_HSP7D.4.8	*Corbicula fluminae*	0.0	0.0	80%	81%
Metallothionein (isoform 1)	Dp_MT.6.6	Db_MT.6.6	*Meretrix meretrix*	6e-05	6e-05	80%	80%
mTOR	Dp_LOC105331599	Db_LOC105331599	*Crassostrea gigas*	0.0	0.0	72%	72%
Na/K ATPase	Dp_LOC106058320	Db_LOC106058320.1.2	*Tridacna squamosa*	0.0	0.0	76%	77%
Succinate dehydrogenase	Dp_LOC101864456.1.3	Db_LOC105338659	*Aplysia californica*	9e-14	2e-16	66%	67%
Acid phosphatase	Dp_LOTGIDRAFT_139839	Db_contig_19914	*Pomacea canaliculata*	1e-11	0.0	67%	95%
Thioredoxin reductase 1	Dp_LOC105322705.2.2	Db_LOC105322705	*Crassostrea spp*.	0.0	0.0	71%	71%
MRP1 (Abcc1 gene)	Dp_LOC105347802.1.2	Db_LOC105347802	*Pomacea canaliculata*	0.0	0.0	69%	69%
MDR1 (Abcb1 gene)	Dp_LOC101858982.2.4	Db_LOC101858982.2.2	*Ruditapes philippinarum*	0.0	0.0	68%	70%

*D*. *polymorpha* and *D*. *rostriformis bugensis* databases were used to find common ecotoxicological biomarkers. Contig coding sequences were then screened in NCBInr (*blastn*) and closest species (excluding *D*. *polymorpha* and *D*. *rostriformis bugensis*) were reported according with E-values and identity percentages. GABARAP: Gamma-aminobutyric acid receptor-associated protein; mTOR: mechanistic target of rapamycin; MRP1: Multidrug resistance-associated protein 1; MDR1: Multidrug resistance protein 1; Cds: coding sequence.

**Table 9:** Interspecies sequence homologies.

Biomarkers	*D. polymorpha*		*D. rostriformis bugensis*		Interspecies Cds homology		
	Contig accession	Cds length	Contig accession	Cds lengt	% Ident.	E-val.	Gap
Acetylcholinesterase	Dp_LOC105324424.2.2	1,599	Db_LOC105324424.3.3	1,644	91%	0.0	0 (0%)
Beta Actine	Dp_ACT.1.3	1,128	Db_ACT.2.4	1,128	98%	0.0	0 (0%)
Catalase	Dp_LOC105339902	1,515	Db_LOC105339902.2.4	1,515	91%	0.0	0 (0%)
Superoxide dismutase (Cu-Zn)	Dp_SODC	459	Db_SODC	459	91%	0.0	0 (0%)
Superoxide dismutase (Mn)	Dp_LOC101852344	621	Db_LOC101852344	621	92%	0.0	0 (0%)
Estrogen receptor	Dp_LOC105318922.2.2	1,476	Db_LOC105318922.1.2	1,476	85%	0.0	111 (3%)
GABARAP	Dp_LOC105335545.1.2	351	Db_LOC105335545.1.6	351	95%	7e-164	0 (0%)
Glutathione peroxidase 1	Dp_GPX1.1.2	426	Db_GPX1.1.2	426	94%	0.0	0 (0%)
Glutathione peroxidase (Se)	Dp_LOC106070504.1.5	729	Db_LOC106070504.6.8	714	85%	0.0	15 (2%)
Heat Shock Protein70	Dp_HSP7D.3.8	1,959	Db_HSP7D.4.8	1,965	92%	0.0	14 (<1%)
Metallothionein (isoform 1)	Dp_MT.6.6	219	Db_MT.6.6	219	100%	6e-117	0 (0%)
mTOR	Dp_LOC105331599	7,422	Db_LOC105331599	7,410	92%	0.0	12 (<1%)
Na/K ATPase	Dp_LOC106058320	3,093	Db_LOC106058320.1.2	3,096	89%	0.0	3 (<1%)
Succinate dehydrogenase	Dp_LOC101864456.1.3	504	Db_LOC105338659	504	93%	0.0	0 (0%)
Acid phosphatase	Dp_LOTGIDRAFT_139839	1,293	Db_contig_19914	1,098	90%	0.0	5 (0%)
Thioredoxin reductase 1	Dp_LOC105322705.2.2	1,788	Db_LOC105322705	1,932	92%	0.0	3 (<1%)
MRP1 (Abcc1 gene)	Dp_LOC105347802.1.2	3,510	Db_LOC105347802	4,686	91%	0.0	6 (<1%)
MDR1 (Abcb1 gene)	Dp_LOC101858982.2.4	4,032	Db_LOC101858982.2.2	4,023	89%	0.0	15 (<1%)

Coding sequence from *D*. *polymorpha* and *D*. *rostriformis bugensis* biomarkers contigs were used for interspecies homology analysis. Length of coding sequences were also indicated (in base pair; For acronym significations, see Table 8).

By providing *Dreissena* sequences through a user-friendly interface, we open the way to further explorations of Dreissenids molecular mechanisms by such biomarker assays development, primer design allowing targeted expression analysis or promising proteogenomics studies when coupling with mass spectrometry analysis.

## Supplementary information

Figure S1.

Table S1.

## Data Availability

Parameters to involved softwares tools are described in the following paragraph. **DRAP** (*De novo* RNA-seq Assembly Pipeline): version 1.7, code available online at http://www.sigenae.org/drap/quick_start.html. **BLAST**: version 2.2.26, e-value under 1e-5 parameter. **RNAmmer**: version 1.2, standard parameters. **RepeatMasker**: version open-4-0-3, -engine crossmatch -gccalc -species *Crassostrea gigas* parameters. **InterProScan**: version 4.8, -goterms -pathways parameters. **BWA**: version 0.7.12, standard parameters, mem algorithm. **SAMtools view**, **sort and index programs**: version 1.1, standard parameters. **SAMtools IdxStats program**: version 1.1, standard parameters. **SAMtools rmdup program**: version 1.1, standard parameters. **GATK**: version v3.0-0-g6bad1c6, -glm BOTH parameter. **BLAT**: version 35 × 1, standard parameters. **BUSCO**: version 3.0.2 using the metazoa OrthoDB (v. 9), standard parameters.
