# Supplementary material for: Environmental transcriptomes of invasive dreissena, a model species in ecotoxicology and invasion biology
Source: Sci Data. 2019 Oct 25;6:234. doi: 10.1038/s41597-019-0252-x (PMC6814772; doi:10.1038/s41597-019-0252-x)
Supplement: Supplementary file 1 — Figure S1. [file 41597_2019_252_MOESM1_ESM.pdf]

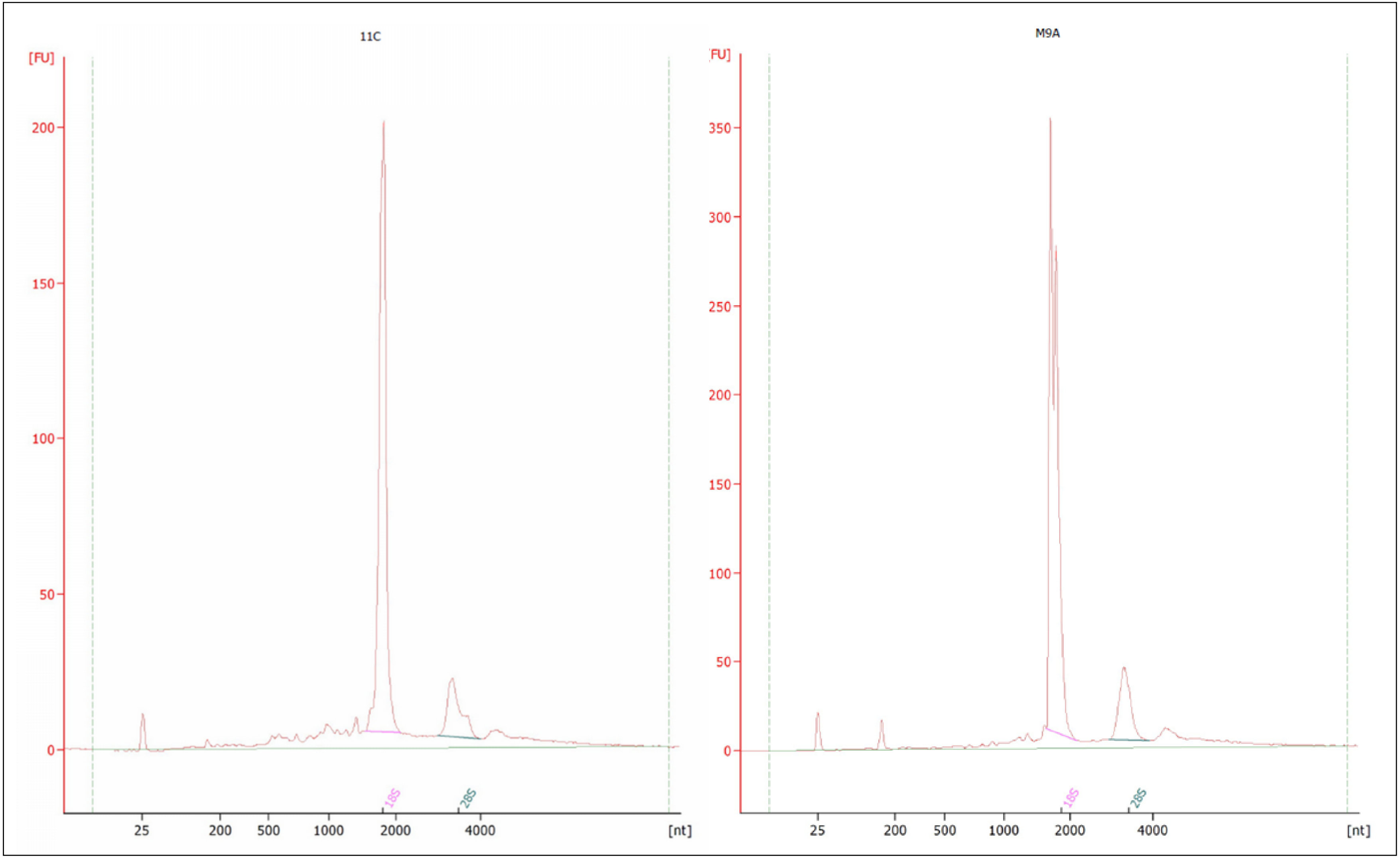

Figure S1: Representative electrophoregram of *D. rostriformis bugensis* (sample 11C) and *D. polymorpha* (sample M9A)
