# Supplementary material for: Environmental transcriptomes of invasive dreissena, a model species in ecotoxicology and invasion biology
Source: Sci Data. 2019 Oct 25;6:234. doi: 10.1038/s41597-019-0252-x (PMC6814772; doi:10.1038/s41597-019-0252-x)
Supplement: Supplementary file 2 — Table S1. [file 41597_2019_252_MOESM2_ESM.pdf]

Table S1 – Sediment contamination and site localisation. For each site, a sample site code was assigned to name raw reads (*e.g.* 1*.fastq or 3p*.fastq from Riaucourt and Scey, respectively). Geographic coordinates are indicated in decimal degrees.

Sites		Riaucourt	Scey	Seurre	Nantua	Oise	Courcelles	Condé	Saint-Mihiel	Madine	Jouy-aux-Arches	Montigny1	Montigny2
**Site topography**		canal	harbor	harbor	lake	canal	harbor	pond	canal	lake	canal	canal	canal
**Sample site codes**		1	2	3p	4	P5	6	7p	8	9	11	12 and 12p	12pp
**Longitude (DD)**		5.150222	5.974818	5.142649	5.601775	2.509842	3.026757	3.597964	5.534464	5.747214	6.078712	6.161272	6.165338
**Latitude (DD)**		48.1793889	47.653304	47.001986	46.156036	49.280340	50.425258	50.459077	48.887146	48.929616	49.065957	49.108543	49.112834
**Anthracene**	µg.kg^{-1} dry matter	390	10	27	513	122	1304	334	48	42	114	32	841
**Benzo(a)anthracene**		2890	46	190	2362	218	5007	1108	214	487	494	175	3324
**Benzo(a)pyrene**		2600	52	248	2253	203	5291	920	82	425	503	214	3380
**Benzo(ghi)perylene**		1570	48	289	1280	133	2770	609	210	239	306	184	1785
**Benzo(k)fluoranthene**		1490	27	140	1211	127	3073	564	163	198	232	130	1920
**Chrysene**		2560	25	217	2293	204	5310	1102	230	482	492	159	3687
**Fluoranthene**		5909	90	509	4586	448	12072	2065	737	940	1142	425	7762
**Indenolpyrene**		955	14	79	791	70	1607	334	130	150	172	159	1140
**Naphtalene**		81	5	5	5	11	130	182	18	5	5	142	1043
**PCB101**		2.5	2.5	2.5	2.5	2.5	2.5	9	2.5	2.5	2.5	2.5	2.5
**PCB118**		2.5	2.5	2.5	2.5	2.5	2.5	12	2.5	2.5	2.5	2.5	2.5
**PCB138**		2.5	2.5	2.5	2.5	2.5	2.5	2.5	2.5	2.5	2.5	2.5	2.5
**PCB153**		2.5	2.5	2.5	2.5	2.5	2.5	2.5	2.5	2.5	2.5	2.5	2.5
**PCB180**		2.5	2.5	2.5	2.5	2.5	2.5	2.5	2.5	2.5	2.5	2.5	2.5
**PCB28**		2.5	2.5	2.5	2.5	2.5	2.5	7	2.5	2.5	2.5	2.5	2.5
**PCB52**		2.5	2.5	2.5	2.5	2.5	2.5	11	2.5	2.5	2.5	2.5	2.5
**Phenanthrene**		1910	25	191	1687	148	4579	1620	203	223	489	175	2606
**Sum PCB**		2.5	2.5	2.5	2.5	2.5	2.5	44	2.5	2.5	2.5	2.5	2.5
**Al**	mg.kg^{-1} dry matter	25840	49250	43790	4338	15920	23070	84850	29580	8708	65680	67210	40840
**As**		22.5	24	15.4	4.5	3.6	16.2	8.2	7.8	21.9	26.4	24.1	11.4
**Cd**		0.3	0.2	0.8	0.2	0.1	54.7	0.4	0.3	0.2	1	0.9	0.3
**Cr**		116.8	58.3	59.7	42.2	46.5	62.4	122	53.9	23	93.3	93.9	81.5
**Co**		7.7	8	8.1	2.5	3.9	6.1	17.9	7.2	5.4	13.3	11.6	6.8
**Cu**		16.5	13	108.5	39.8	12.1	79	58.3	23.1	5.9	44.1	38.8	44.9
**Fe**		42200	19130	22900	6848	11150	14740	25250	17230	24230	40480	33660	27660
**Mn**		235.4	188.1	372.9	126.4	258.2	346.3	695.8	204.2	336.5	692.9	626.8	882.3
**Hg**		0.05	0.01	0.14	0.52	0.07	4.46	0.16	0.06	0.02	0.2	0.2	0.04
**Ni**		28	16	23.6	21.7	8.6	17.6	49	22.6	10.6	42	37.1	16
**Pb**		25.2	24.3	34.8	143.5	15.7	1004	30.4	18.6	8.5	61.8	58.5	69.7
**Zn**		129.1	48.3	265	128	60.2	2443	192.8	122.5	41.3	275.9	214.4	109.9
